# Chest-CT mimics of COVID-19 pneumonia—a review article

**DOI:** 10.1007/s10140-021-01919-0

**Published:** 2021-03-01

**Authors:** Eleonora Carlicchi, Pietro Gemma, Antonio Poerio, Antonella Caminati, Angelo Vanzulli, Maurizio Zompatori

**Affiliations:** 1 Post-graduate School in Radiodiagnostic, Università degli Studi di Milano, Milan, Italy; 2 Radiology Unit, Santa Maria della Scaletta Hospital, Imola, Italy; 3 Respiratory Medicine and Semi-Intensive Therapy Unit, Respiratory Physiopathology and Pulmonary Haemodynamics Services, San Giuseppe Hospital Multimedica, Milan, Italy; 4 Radiology Unit, ASST Grande Ospedale Metropolitano Niguarda, Piazza Ospedale Maggiore 3, 20162 Milan, Italy; 5 Oncology and Hemato-Oncology Unit, Università degli Studi di Milano, via Festa del Perdono 7, 20122 Milan, Italy; 6 Radiology Unit, San Giuseppe Hospital Multimedica IRCCS, Milan, Italy

**Keywords:** COVID-19 pneumonia, Chest HRCT, Differential diagnosis

## Abstract

Coronavirus disease 2019 (COVID-19) emerged in early December 2019 in China, as an acute lower respiratory tract infection and spread rapidly worldwide being declared a pandemic in March 2020. Chest-computed tomography (CT) has been utilized in different clinical settings of COVID-19 patients; however, COVID-19 imaging appearance is highly variable and nonspecific. Indeed, many pulmonary infections and non-infectious diseases can show similar CT findings and mimic COVID-19 pneumonia. In this review, we discuss clinical conditions that share a similar imaging appearance with COVID-19 pneumonia, in order to identify imaging and clinical characteristics useful in the differential diagnosis.

## Introduction

Coronavirus disease 2019 (COVID-19) was first reported in Wuhan, China, in early December 2019 as an acute lower respiratory tract infection [1] with subsequent rapid outbreak worldwide, being declared pandemic in March 2020.

Currently, WHO estimates report that there have been 42.966.344 confirmed cases and 1.152.604 confirmed deaths due to COVID-19 worldwide, with 9.472.859 confirmed cases in Europe and 525.782 cases in Italy [2].

COVID-19 clinical presentation is highly variable and nonspecific, with fever, cough, dyspnea, anosmia, dysgeusia, fatigue, and muscle aches being the most common symptoms. Some cases progress to a severe viral pneumonia with respiratory failure and even death, while others recover completely. Moreover, COVID-19 infection can be asymptomatic in a significant number of cases.

The standard diagnostic method for COVID-19 infection is the reverse-transcription polymerase chain reaction (RT-PCR), which detects virus nucleotides in oropharyngeal and nasopharyngeal swab samples, bronchoalveolar lavage or tracheal aspirate.

RT-PCR sensitivity is estimated between 60 and 71% [3], probably due to error sampling, specimen type, and viral load at the time of examination. RT-PCR limitations include sample collection and transportation, kit shortage, and processing period.

Chest-computed tomography (CT) is a rapid and widely used diagnostic tool for thoracic pathology, including lung infectious and non-infectious diseases. Some studies report that chest-CT may show pulmonary abnormalities in COVID-19 patients with a false-negative RT-PCR test in the early stages of disease [4, 5], and can also identify features compatible with COVID-19 pneumonia, in asymptomatic patients undergoing CT examination for other reasons in the setting of community transmission.

Chest-CT has been widely used during the COVID-19 pandemic, being a fast and easily accessible technique in most healthcare centers. CT has a high sensitivity, about 94–97% [6, 7], in detecting early signs of COVID-19 pneumonia, disease progression, complications, and possible alternative diagnoses such as heart failure or pulmonary embolism. However, the specificity of CT is low (about 37%) [6], since many lung diseases that can mimic COVID-19 pneumonia CT appearance. Indeed, many radiology professionals societies recommend against performing CT as a primary technique for the diagnosis of COVID-19 pneumonia [8].

Particularly, the Fleischner Society Multinational Consensus Statement attempts to standardize the criteria for the use and interpretation of CT in COVID-19 pandemic, by stratifying patients according to risk and severity of symptoms [9]. According to the Multinational Consensus Statement [9], CT is not recommended as a screening tool for COVID-19 infection in asymptomatic patients nor in patients with mild symptoms compatible with COVID-19 infection, unless there are risk factors for progression. CT is indicated in patients with moderate to severe symptoms, regardless of the RT-PCR result, and in patients with confirmed COVID-19 infection with worsening respiratory symptoms.

Nowadays, in most institutions, pulmonary CT is usually performed using 64-, 256-, or 128-slice multidetector row CT scanners. Images are acquired with the use of standard tube voltage and current settings (120 kV, 200 mAs, varying depending on patients’ body mass index, 16 × 0.75 collimation and pitch = 0.938) with a scanning range covering the area from lungs apices to the diaphragm, generally with a cranio-caudal scanning direction.

Patients are generally scanned in supine position during a deep-inspiration breath hold. However, prone position can be particularly useful to differentiate between parenchymal abnormalities in the posterior regions and dependent lung atelectasis. Expiratory acquisition may be needed to distinguish air-trapping from hypo-vascular mosaic attenuation; it needs to be noted though, that expiratory acquisitions are not routinely performed as it might be physically demanding for patients with acute disease, such as COVID-19 pneumonia.

Images are reconstructed with a slice thickness of 1.00 or 1.25 mm and photographed at window settings levels appropriate for the examination of lung parenchyma (window level, − 600 to – 700 HU; window width, 1200–1500 HU) and the mediastinum (window level, 20–40 HU; window width, 400 HU).

The key imaging finding in COVID-19 pneumonia is ground-glass opacity (GGO), defined as an area of hazy increased lung attenuation which do not conceal bronchial and vascular structures [10]. It represents the partial filling of alveolar airspaces and may be due to countless different causes so its diagnostic value in isolation is scarce. Causes of GGO may be usefully divided into vascular (i.e., increased capillary blood volume or pressure such as in case of cardiac insufficiency) and non-vascular (i.e., partial filling or collapse of alveoli, and interstitial thickening due to fluid, cells or fibrosis) or a combination of these.

**Fig. 1:**
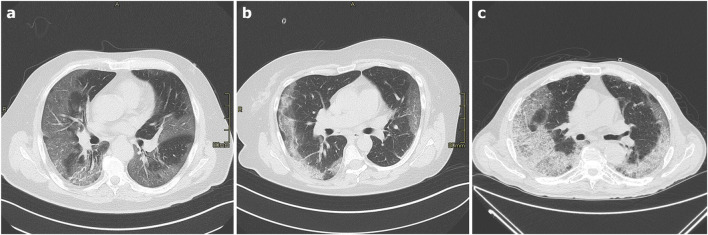
**a**–**c** Axial HRCT images of three different patients with COVID-19 pneumonia (**a**–**c**) showing bilateral, patchy ground-glass parenchymal opacities with prevalent peripheral and mid-lower lobes distribution

**Fig. 2:**
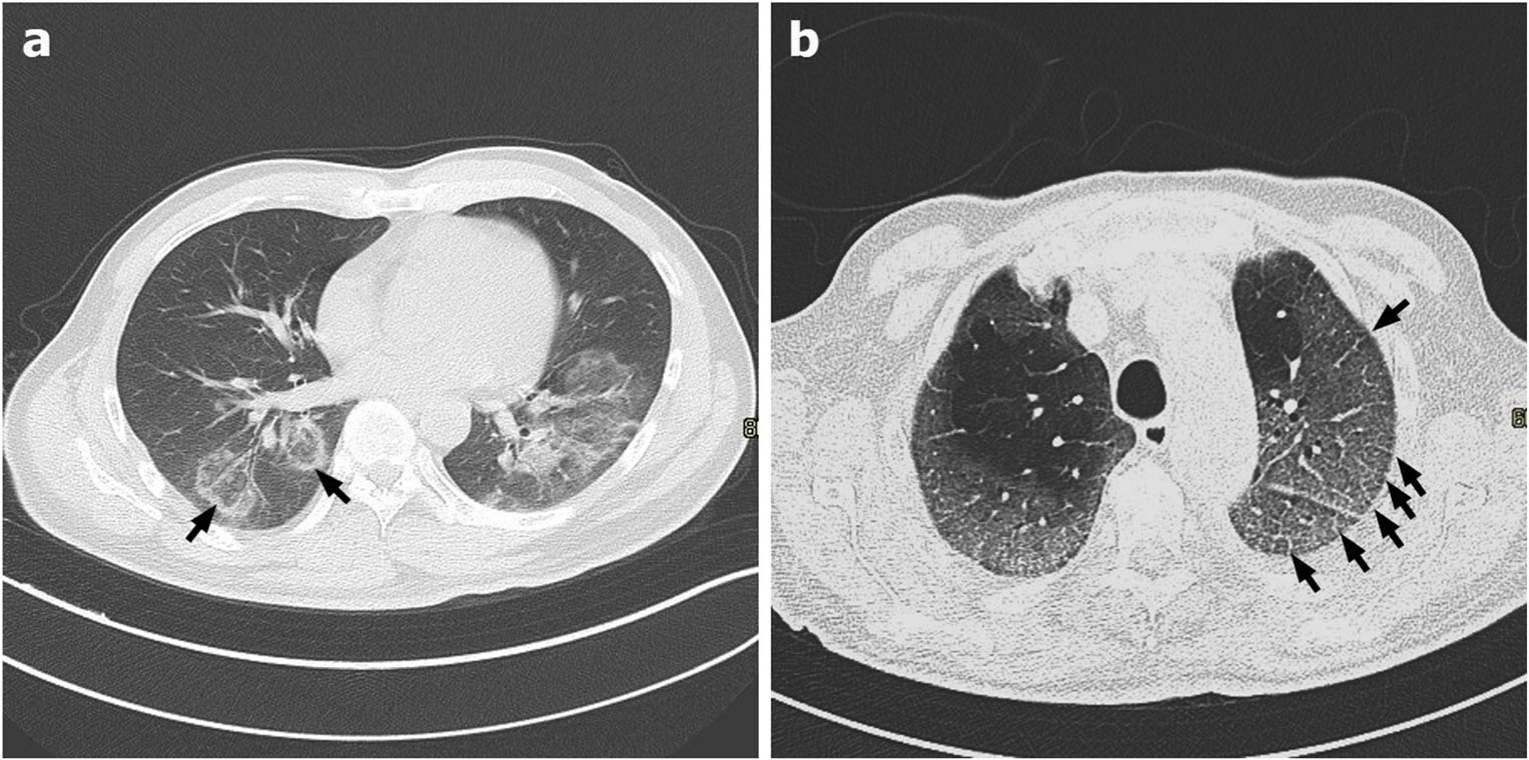
**a**–**b** Axial HRCT images of two patients with COVID-19 pneumonia showing an organizing pneumonia pattern (**a**), with bilateral ground-glass opacities, lower lobes prevalent distribution, and the classic reverse halo sign, or atoll sign (black arrows in **a**), in the right lower lobe; and a “crazy paving” pattern (**b**) resulting from ground-glass opacities superimposed to interlobular septal thickening (black arrows in **b**)

The most frequent CT findings in COVID-19 pneumonia are bilateral, multifocal, patchy GGO, with or without concurrent areas of consolidation, typically with a basal peripheral distribution [8] (Fig. 1). Reticular and/or interlobular septal thickening can also be seen, superimposed on the GGO, resulting in a “crazy-paving pattern” [9, 11, 12] (Fig. 2). Pure consolidation is uncommon and found in elder patients (> 50 years), in progressive cases and more severe disease [1]. In patients developing an organizing pneumonia (OP) pattern, CT may show the “reverse halo sign” (an irregular opacity with a dense peripheral ring of consolidation and a central area of GGO), which was reported in 32,1% of patients with moderate disease and in 13% of more severe cases [13] (Fig. 2).

COVID-19 infection is associated with microvascular thrombosis, and an increased frequency of pulmonary embolism and pulmonary infarctions, visualized on CT as peripheral opacities [14].

CT may also help differentiate other lung diseases, acute heart failure, and, when performed with contrast medium, pulmonary thromboembolism [9].

CT manifestations of COVID-19 pneumonia may widely vary also depending on the phase and on the severity of the disease, and in 10.6% of symptomatic patients chest-CT can be normal [15].

The aim of this essay is to present a set of pathological conditions that can mimic COVID-19 pneumonia based on the similarities of CT appearance, and to describe imaging features and clinical characteristics that can be helpful in the differential diagnosis.

## Pulmonary infections

### Bacterial pneumonia

Lung infections are common in clinical practice and bacterial pneumonia is one of the most frequent causes. It is classified as community acquired pneumonia (CAP), aspiration pneumonia or nosocomial pneumonia (NP) based on clinical scenario [16].

CAP is commonly caused by *Streptococcus pneumoniae* and *Mycoplasma pneumoniae*; *Staphylococcus aureus* and Gram-negative (Enterobacteriae or *E. coli*) often cause NP, while aspiration pneumonia is due to microorganisms found in oropharynx such as Gram-negative cocci.

Clinical manifestations are fever, chills, cough, and sometimes chest pain; symptoms can be milder or absent in immunocompromised patients [17].

When pneumonia is suspected, diagnosis is confirmed by imaging evaluation, with chest X-ray (CXR) being the most used tool. CT is more sensitive but usually performed in unclear cases or when complications are suspected [16].

Imaging findings are variable: in CAP, CT images classically show airspace consolidation and can also evidence GGO, centrilobular nodules and bronchial walls thickening, with or without pleural effusion. Rarer presentations are round and multi-lobar pneumonia. The first, mainly seen in children, is typically characterized by a focal, round-shaped consolidation at imaging. Round pneumonia should be suspected in case of a rapid growing pulmonary nodule/mass, which disappears after antibiotic therapy [18].

Multi-lobar or bilateral pneumonia are non-focal patterns of CAP, with greater extent, often seen in patient with an underlying lung disease causing parenchymal distortion [17, 19].

Aspiration pneumonia is secondary to inhalation of oropharyngeal or gastric secretions into the lower respiratory tract. Imaging generally shows bilateral and peri-hilar air-space consolidations, involving lower lobes, especially in the right lung [20].

NP is a complication of hospitalized patients defined as a pneumonia occurring 48 h after the admission or 48 h after hospital discharge [21]. Imaging findings are bilateral, diffuse, or multiple foci of consolidation involving more than one lobe, frequently associated with pleural effusion [22].

Complications may occur in any pneumonia but are more frequent in CAP and NP, especially in immunocompromised patients, and are usually detected by CT. Lung cavitation suggests bacterial etiology, with *S. aureus* and anaerobic bacteria being the most frequent agents in immunocompetent patients, and Aspergillus in immunocompromised ones. Pleural effusions are frequent during uncomplicated CAP and are reactive in nature, resolving after antibiotic therapy, 5–10% of these might complicate and progress to empyema [16].

### Viral pneumonia

Viruses are the main cause of acute respiratory infections. Viral pneumonia can be caused by different viruses depending on patient’s age and immune status. Clinical symptoms are non-specific and depend on immunological status (being more severe in immunocompromised patients), age, and virus’s prevalence [23].

Imaging features are variable and non-specific since they overlap and are shared with other non-viral lung diseases. CT findings reflect the pathogenesis of viral infection: viruses of the same family, sharing similar pathogenesis, generally show similar CT appearance [23].

Adenovirus manifests as a mild upper respiratory tract infection in most immunocompetent patients. However, it can also cause acute bronchiolitis and evolve to bronchopneumonia. CT findings in acute adenovirus pneumonia are bilateral, multifocal GGO with patchy consolidations, often with a segmental-lobar distribution, suggestive of bronchopneumonia, associated with tree-in-bud-opacities [23]. Sometimes, expiratory acquisitions may demonstrate areas of air trapping, visualized as areas of decreased attenuation of lung parenchyma during expiration, due to small airway obstruction and reduced lung compliance. They are usually related to chronic bronchiolitis.

Cytomegalovirus (CMV) pneumonia is more frequent in immunocompromised patients, such as HIV patients, patients on long-term corticosteroid therapy or organ transplant receivers, especially in the early phase (30–100 days) after transplantation. Immunocompetent patients are usually asymptomatic.

CT in CMV pneumonia demonstrates bilateral and asymmetric GGO, associated with random distributed, poorly defined pulmonary nodules and air-space consolidation. Interlobular septal thickening can be observed [23, 24].

Influenza viruses are a diffuse group of infective agents (influenza virus A, B, C, and D) causing seasonal epidemic, or pandemic, upper respiratory tract infections. Infections are usually mild and self-limited but, in immunocompromised patients, children or elderly people, pneumonia may occur, mostly caused by Influenza A virus.

**Fig. 3:**
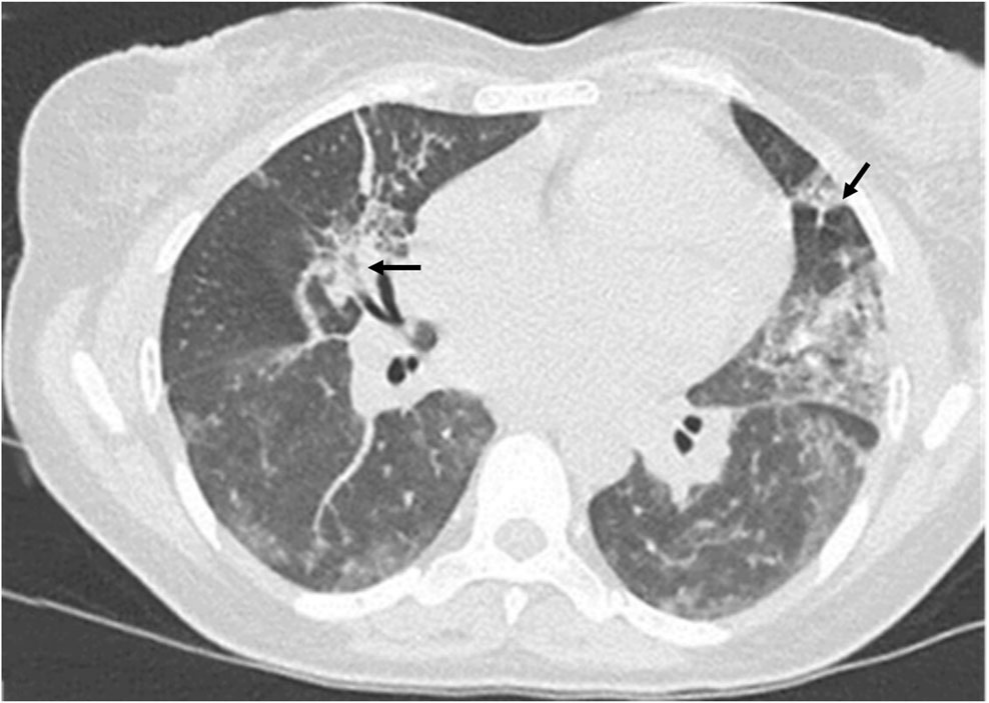
Axial HRCT scan of a patient with H1N1 pneumonia shows bilateral patchy ground-glass opacities associated with small areas of consolidation (black arrows)

CT can show focal or diffuse, bilateral GGO, associated with consolidations that tend to be confluent (Fig. 3). Bronchiectasis and small centrilobular nodules are frequently observed in Influenza pneumonia while are rare in COVID-19 [25]. During influenza pneumonia, pleural effusion and cavitation can develop [25, 26].

Human metapneumovirus (HMPV) causes approximately 4% of CAP in winter. Immunocompetent patients generally recover with no sequelae, while immunocompromised patients may develop a severe, life-threatening pneumonia. HMPV infection causes alveolar damage with mucous plugging, resulting in a CT pattern of branching centrilobular nodules and GGO [23].

Human coronaviruses are an important group of pathogens that cause upper and lower acute respiratory tract infections, and acute respiratory distress syndrome. SARS and MERS viruses belong to this group.

Imaging features of SARS pneumonia resemble those observed in other CAP: most common findings are airspace consolidations with unifocal pattern being more frequent than the multifocal one. Parenchymal involvement is predominately in the lower lobes with peripheral distribution.

MERS pneumonia is a severe condition that can lead to respiratory failure more rapidly than SARS pneumonia does. CT in MERS pneumonia usually demonstrates multifocal patchy nodular consolidations with extensive GGO involving basal and subpleural regions of both lungs [12, 27].

CT features of COVID-19 pneumonia can widely overlap with those observed in other viral pneumonias. However, some findings have been reported to be more indicative of COVID-19 pneumonia rather than other etiology and can help to differentiate this entity.

Pure GGO or mixed GGO and consolidation patterns are the most typical in COVID-19 pneumonia, while pure consolidations are rarely seen and more common in other viral pneumonia such as influenza pneumonia.

COVID-19 infection generally causes bilateral large parenchymal lesions (> 5–10 cm), often round-shaped, involving multiple lobes, with a characteristic peripheral distribution.

Interlobular septal thickening, bronchial wall thickening, linear opacities, and vascular enlargement are other common findings. “Crazy paving” pattern is more frequent in COVID-19 pneumonia compared to other viral infections, particularly influenza [25].

Conversely, tree-in-bud opacities, multiple nodules, bronchiectasis, extensive air-space consolidations, pleural effusions, and lymph node enlargement are unusual in COVID-19 pneumonia and more suggestive of other agents [12, 26, 27]. Cavitations are unusual in COVID-19 infections and suggestive of bacterial etiology [28].

In severe cases, requiring assisted ventilation, diffuse lung opacities due to diffuse alveolar damage (DAD) or OP can be observed. Currently, little has been written about the long-term sequelae of COVID-19 pneumonia and further studies are required.

### Pneumocystis jiroveci pneumonia (PJP)

Pneumocystis jiroveci is an atypical fungus that can cause life-threatening infections in immunocompromised patients, especially in case of cell-mediated immunity deficiency [29].

PJP is among the most frequent opportunistic infections in HIV patients, particularly when the CD4+ cells count are <200 cell/mm3 [24, 29].

Onset is gradual with non-specific signs and symptoms, such as fever, dry cough, and dyspnoea which can last up to 1 month [30].

In some cases, PJP can lead to acute respiratory distress syndrome (ARDS) with severe hypoxemia and necessity of mechanical ventilation [29]. PJP rarely affects non-HIV patients, but, when it does, it shows a more aggressive course leading to respiratory failure, due to the severe inflammatory response [31].

CT is the most used imaging tool to evaluate pneumonia in immunocompromised patients.

**Fig. 4:**
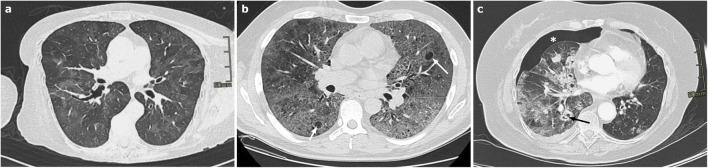
**a**–**c** Axial HRCT images of three patients with different stages of Pneumocystis jiroveci pneumonia showing, at an early stage (**a**), bilateral, diffuse ground-glass opacities with a mosaic attenuation pattern; and in advanced stages (**b**–**c**) more extensive ground-glass opacities and parenchymal, variable in shape and size cysts (arrows in **b** and **c**), whose rupture can sometimes lead to pneumothorax (white * in **c**)

The main findings of PJP are extensive, homogeneous symmetric GGO, with a central and mid-upper lobe distribution and relative sub-pleural sparing, although diffuse pattern sometimes occurs. In more advanced stages, a “crazy paving” pattern can be seen. A mosaic attenuation pattern is described in about 50% of cases [29] (Fig. 4).

Lung consolidations are more common in rapidly progressive cases in non-HIV patients, reflecting the parenchymal damage secondary to the host immune response [24, 29].

Lung air-containing cysts, variable in shape and size, are present in 1/3 of patients and are more frequent in HIV patients. Cysts are associated with a higher risk of pneumothorax and can resolve after appropriate infection treatment.

Sometimes, a granulomatous inflammation may develop as solitary or multiple nodules ranging from few millimeters to 1 cm. Architectural distortion and residual fibrosis may persist after recovery [24, 29].

## Hypersensitivity pneumonitis (HP)

HP is an inflammatory and/or fibrotic disease affecting lung parenchyma and small airways, provoked by an immune reaction in susceptible individuals after repeated exposure to one or more inciting agents [32].

Historical classification of HP in acute, subacute, and chronic has long since been abandoned [33].

The latest proposed classification is based on the degree of radiological and/or histopathological fibrosis and divide HP in fibrotic (i.e., presence of fibrosis with or without inflammation) or nonfibrotic (i.e., purely inflammatory) [32].

This new approach is easier to apply and better reflects the disease’s clinical course, being parenchymal fibrosis the principal prognostic factor [34, 35].

The imaging features that might mimic COVID-19 pneumonia, mainly concern the non-fibrotic HP, whose main findings are bilateral and symmetric patchy ground-glass areas and consolidations, associated with mosaic pattern attenuation.

**Fig. 5:**
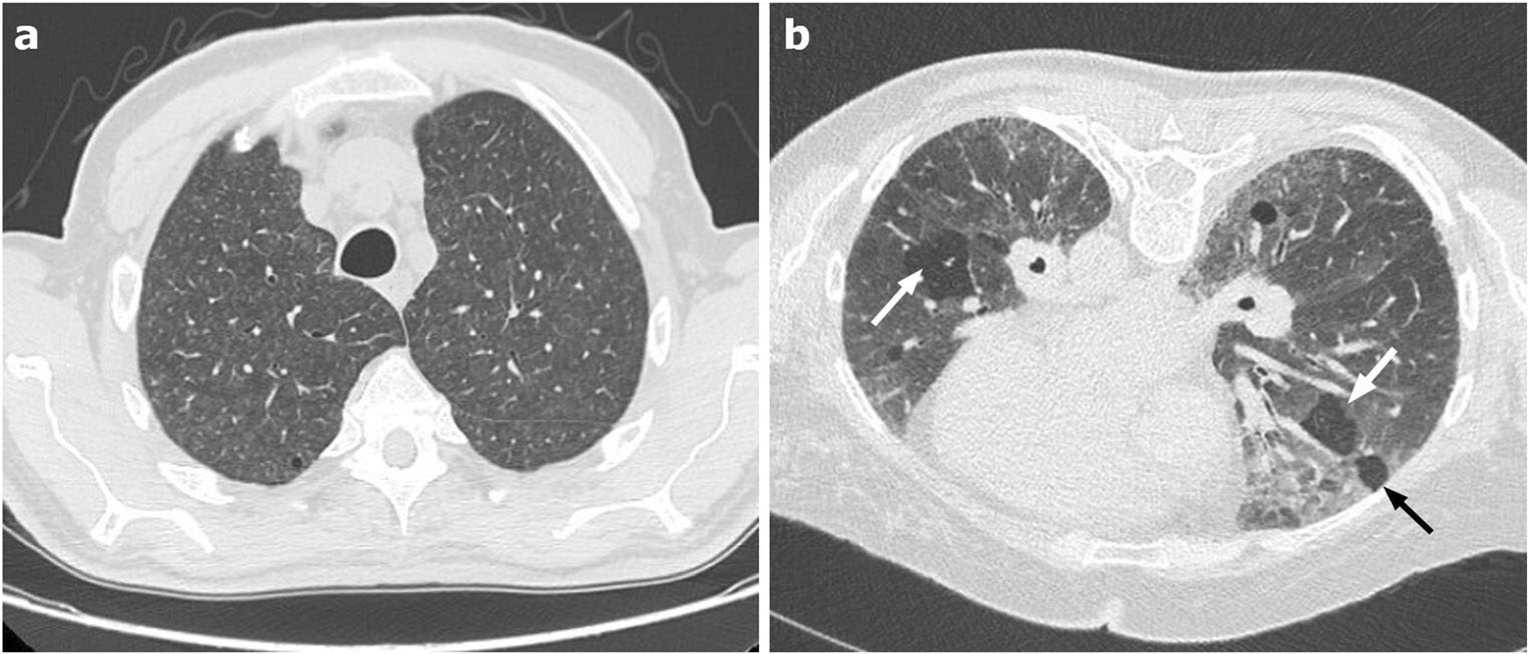
**a**–**b** Axial supine (**a**) and prone (**b**) HRCT images of a patient with non-fibrotic hypersensitivity pneumonitis showing small bilateral ground-glass centrilobular nodules (**a**) and bilateral patchy ground-glass opacities with lobular air-trapping (arrows in **b**) resulting in a mosaic pattern attenuation

However, in non-fibrotic HP, parenchymal alterations are more diffusely distributed, without a definite cranial-caudal or axial gradient; diffuse and bilateral ill-defined, centrilobular nodules are often observed (representing small airway involvement) as well as significative air trapping, which can be confidently identified only by expiratory acquisitions (Fig. 5). These characteristics are unusual in COVID-19 and may be useful, together with the history of antigenic exposure, to differentiate the two entities.

## Acute fibrinous and organizing pneumonia (AFOP)

AFOP is a rare and little-known interstitial lung disease, associated with acute lung injury and poor prognosis, first described in 2002 by Beasley et al. [36].

The distinctive histopathological features are intra-alveolar fibrin deposits (fibrin “balls”) and the presence of OP, while hyaline membranes of DAD and eosinophils are absent [37].

AFOP may be idiopathic or secondary to infections, collagen vascular diseases, hematological malignancy, lung transplantation, drugs, and toxic exposure [38, 39].

Two principal clinical patterns have been described: acute, with a rapidly progressive course leading to respiratory failure, and chronic, less aggressive and slower in clinical progression [36, 39].

CT features are variable with no pathognomonic signs: bilateral multifocal ground-glass opacities with basal predominance and “crazy paving” are frequent. Occasionally, diffuse areas of consolidation with broncho-vascular bundles thickening can be present, some of them with the crescentic morphology and a central GGO (reversed halo or atoll sign) typical of OP [36, 40].

## Acute eosinophilic pneumonia (AEP)

Eosinophilic lung disorders represent a group of diffuse parenchymal diseases, characterized by eosinophil infiltration in alveolar spaces and interstitium, with or without peripheral blood eosinophilia [41].

AEP is an acute febrile illness, with a significant morbidity and mortality if not promptly treated [42].

Some cases are idiopathic, but there is a strong relation with cigarette smoking, particularly with changes in smoking habits, (i.e., starting or restarting after cessation) [43], even exposure to passive smoking can cause AEP [44]. The mean age is 30 years old with a male sex predominance [45].

AEP has an acute onset with fever, cough, and dyspnoea which last about 5 days, and can rapidly progress to respiratory failure if not treated [46]. AEP rapidly responds to glucocorticoids with complete resolution, without relapse or withdrawal [47].

Peripheral eosinophilia is uncommon at presentation but is frequent later in the disease course, while BAL eosinophilia (defined as >25% eosinophils on BAL fluid differential cell count) is characteristic of AEP from the beginning [48].

**Fig. 6:**
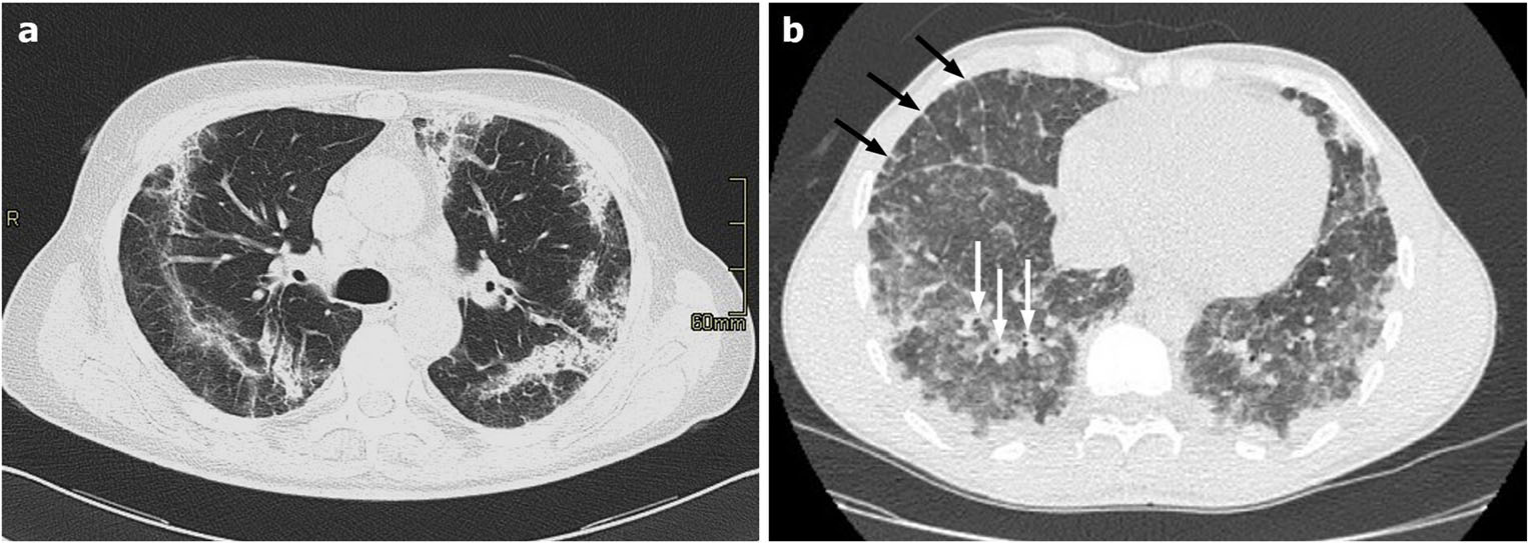
**a–b** Axial HRCT images of two patient with acute eosinophilic pneumonia (**a**–**b**) show parenchymal ground-glass opacities and consolidations with a peripheral and lower lobes prevalent distribution; thickening of interlobular septa (black arrows in **b**) and broncho-vascular bundles (white arrows in **b**) are also seen

CT findings are bilateral patchy GGO, frequently associated with consolidations and smooth interlobular septal thickening, similar to what can be observed in COVID-19 (Fig. 6). These findings usually have no preferential cranio-caudal or axial gradient, but a peripheral and lower lobes prevalent distribution can be observed [49].

Pleural effusions, often bilateral, are typical and seen in >90% of cases, as well as lymph nodes enlargement [48].

## Diffuse alveolar hemorrhage (DAH)

DAH is a clinicopathologic syndrome consisting in diffuse bleeding into alveolar spaces due to an injury to the alveolar capillaries (pulmonary capillaritis) [50].

DAH can appear at any age, without sex predilection and can be secondary to a wide variety of conditions such as systemic vasculitis, connective tissue diseases, infections, coagulation disorders, drug toxicities, or hematopoietic stem cells transplantation (SCT) [50].

Clinical course is unpredictable and variable in severity, ranging from complete resolution to life-threatening progressive forms [50, 51].

The classical clinical sign of DAH, hemoptysis, is absent in one third of cases [52]. Other common clinical features are nonspecific such as fever, cough, dyspnea, and chest pain.

Imaging features are equally nonspecific and can mimic any other acute disease that causes diffuse alveolar filling. Diagnosis often requires BAL, which shows a persistent or increasingly bloodier return from lavage [51].

**Fig. 7:**
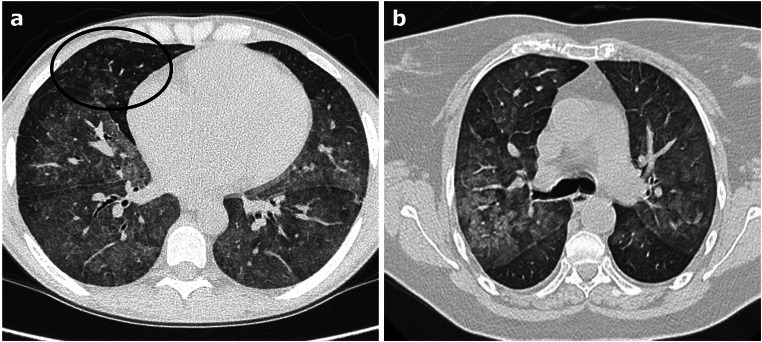
**a**–**b** Axial HRCT images of two different patients with diffuse alveolar hemorrhage show bilateral, diffuse hazy ground-glass (**a**–**b**) and centrilobular opacities (black circle in a) with subtle sub-pleural sparing and a prevalent middle/lower lung zones distribution or “batwing” appearance

In the early acute phase, chest imaging can be normal in 20–50% of cases [50]; when present, common findings are patchy ground-glass, centrilobular opacities without inter-lobular septal thickening, with a prevalent middle/lower lung zones distribution or batwing appearance (Fig. 7). In the subacute phase, interlobular and intralobular septal thickening may develop leading to a crazy-paving pattern.

CT abnormalities resolve in about 2 weeks. In case of recurrent episodes of DAH, fibrotic abnormalities might progressively develop, with architectural distortion and volume loss, coexisting with residual speared lung areas [51].

## Pulmonary edema

Pulmonary edema is a condition characterized by an abnormal accumulation of fluid in lung interstitium and airspaces [53]. Pulmonary edema can be classified according to the pathogenesis in a) hydrostatic pressure edema, due to an increased hydrostatic capillary pressure with left-sided heart failure and fluid overload being the most common causes; b) permeability edema, due to an increased permeability of capillary endothelium, as observed in cases of inhalation injury, infections, aspiration or trauma with or without accompanying ARDS and DAD; and c) mixed pulmonary edema, such as re-expansion or re-perfusion pulmonary edema [53].

Pulmonary edema is a very common finding in clinical practice and hydrostatic edema is reported to be the main non-infectious cause of widespread GGO in hospitalized patients [54].

In hydrostatic edema, common CT features are bilateral GGO, usually symmetric, mostly distributed in the dependent regions of lower lobes and in peri-hilar location. Peri-bronchial cuffing and interlobular septal thickening are also observed, reflecting lymphatic drainage overload [55].

Small bilateral pleural effusions are frequent and help confirming the diagnosis [24, 55].

Permeability edema is caused by changes in capillary epithelium leading to fluid diffusion in presence of normal values of hydrostatic pressure. CT features are similar to those observed in hydrostatic edema, but GGO generally is more confluent, prevalent distribution is usually not in the dependent regions and air-space consolidations are more frequent. Consolidations are prominent in cases of permeability edema associated to ARDS [55]. Conversely, septal thickening and pleural effusion are unusual.

## Pulmonary lymphangitis carcinomatosa (PLC)

Pulmonary lymphangitis carcinomatosa (PLC) is a form of metastatic pattern in advanced malignant tumors, due to the metastatic spreading through the pulmonary lymphatic circle [56]. Tumor spreading into pulmonary lymphatic capillaries hinders lymphatic flow causing accumulation of interstitial and alveolar fluid leading to respiratory dysfunctions [57]. PLC is usually a late complication occurring in patients with a known malignant tumor, more rarely PLC can be the first manifestation of an occult disease. Although it can occur in any malignant disease with chest involvement [56], PLC is frequently associated with adenocarcinomas (80%) [58] with breast cancer (33%), gastric cancer (29%), and lung cancer (17%) being the most common primary tumors [56, 57].

Clinical symptoms are generally progressive dyspnea and dry cough, observed in about half of the patients. PLC is associated with a poor prognosis with an average survival of three month after the onset of respiratory symptoms [57].

CT is the technique of choice for the diagnosis of PLC and should be performed when suspected. CT common features are nodular and irregular thickening of interlobular septa and broncho-vascular bundles with preserved lung architecture (contrary to what observed in fibrotic lung diseases), hilar and mediastinal lymph node enlargement and pleural effusions. Lung infiltrates, GGO or consolidations, can also be observed. These findings tend to be unilateral, commonly on the right side, with prevalent lower lobes involvement [56, 59].

## Transfusion-related acute lung injury (TRALI)

TRALI is the most common cause of hemotherapy-related morbidity and mortality, with an estimated incidence ranging between 0.01% and 0.08% per plasma-containing unit transfused, and a mortality rate of 5–14% [60].

TRALI is an acute non cardiogenic pulmonary edema, generally occurring within 6 h following the transfusion of plasma containing blood products, usually between 30 min and 2 h after transfusion [60, 61].

The pathogenesis of TRALI is not completely clear, but neutrophils have a central role because of their cytotoxic effect on pulmonary endothelium resulting in an increased capillary permeability.

Clinical presentation is non-specific and characterized by cough, dyspnea, fever, hypoxemia, cyanosis, leukopenia, and sometimes respiratory failure.

Imaging features are those of pulmonary edema with CT showing bilateral, extensive GGO with broncho-vascular bundles and interlobular septal thickening; air-space consolidation appear with progressive alveolar filling. Cardiac appearance is normal [60, 61]. Despite non-specific symptoms and imaging features, the anamnestic data of a recent blood product transfusion should suggest this entity.

## Acute exacerbation of interstitial lung diseases (AE-ILDs)

Fibrosing interstitial lung diseases (ILD) are a group of chronic lung diseases with variable course in individual patients.

Episodes of rapid deterioration are quite common and unpredictable as they can occur at any stage during the disease course [62].

Acute exacerbation of ILDs (AE-ILD) is an acute respiratory deterioration (increasing hypoxemia and dyspnea) that can rapidly progress to respiratory failure, typically less than 1 month in duration, accompanied by extensive CT lung abnormalities [63].

AE is a well-known complication of idiopathic pulmonary fibrosis (IPF) but it can occur in many other ILDs, including nonspecific interstitial pneumonia (NSIP), chronic hypersensitivity pneumonitis (CHP), and ILD associated with connective tissue disease [62, 64].

Sometimes, an episode of AE-ILD in a previously healthy patient, can be the first manifestation of an unknown ILD.

AE-ILD episodes are more frequent in winter and spring, suggesting that infections could be trigger factors [63].

In ILDs other than IPF, AE is more frequent in those cases with radiological or histopathological pattern of usual interstitial pneumonia (UIP) [65, 66].

AE represents a life-threatening event associated with a poor prognosis with a short-term mortality of 50% despite therapy [67].

AE-ILD diagnosis is based on clinical and radiological findings, especially CT.

**Fig. 8:**
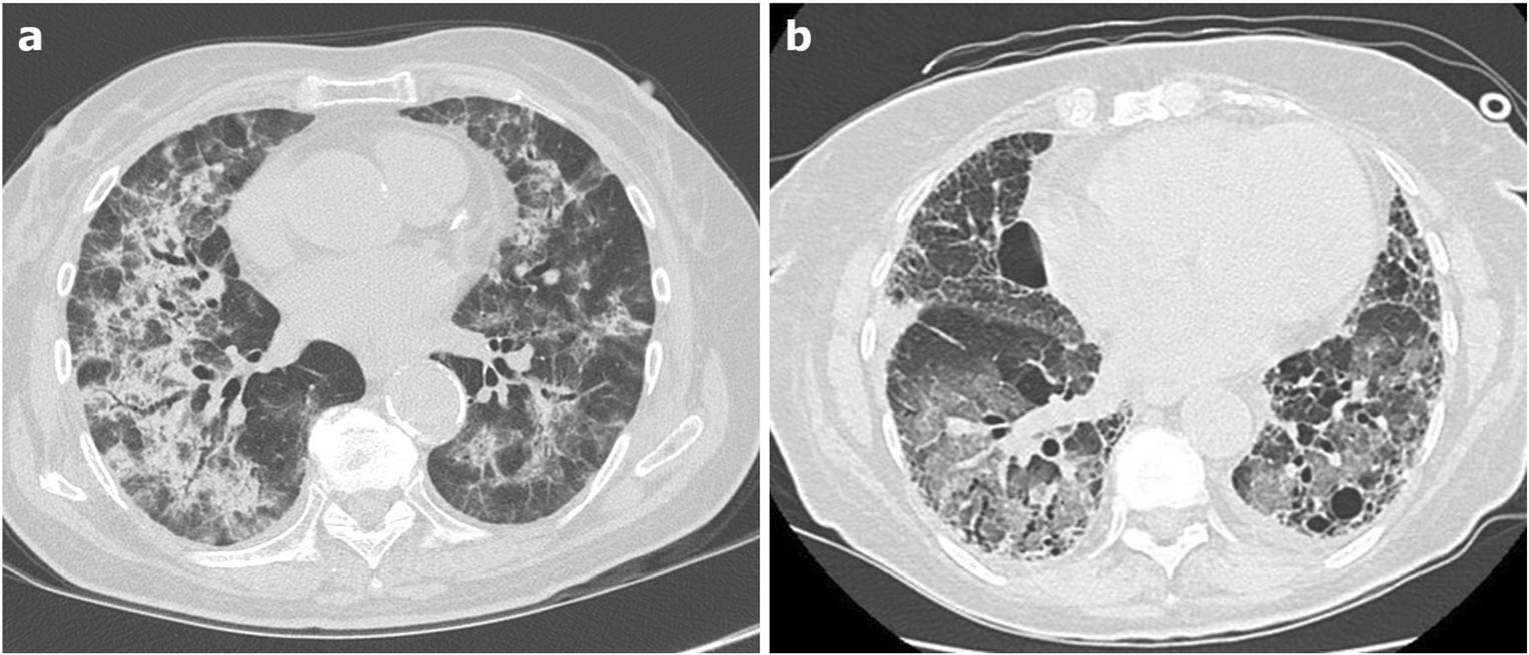
**a**–**b** Axial HRCT images of two patients with acute exacerbation of interstitial lung disease (ILD) show extensive bilateral ground-glass opacities and focal consolidations, superimposed to a background parenchymal pattern consistent with an underlying ILD compatible with non-specific interstitial pneumonia pattern (**a**) and with usual interstitial pneumonia pattern (**b**)

CT in AE-ILD shows new extensive bilateral GGOs, sometimes with focal consolidations, associated to a background pattern consistent with an underlying ILD (often, but not exclusively, UIP) (Fig. 8). Pleural effusion is not common unless cardiac overload coexists [62, 63]. The distribution of the new lung abnormalities is variable and has been classified as peripheral, multifocal, or diffuse [68]; however, the most significant prognostic factor is the extent of the disease on CT, the greater the extension the worse the survival rate [69].

## Electronic cigarette or vaping product use–associated lung injury (EVALI)

Electronic cigarettes (vaping) are devices which aerosolize nicotine and a mixture of chemical components, proposed as a safer alternative to tobacco smoking.

Cases of acute respiratory symptoms associated with CT and histopathologic lung abnormalities have been found in patients with a history of vaping. This suggested that some inhaled components could be harmful, and these cases were referred to as e-cigarette or vaping-product use associated lung injury (EVALI) [70, 71].

EVALI patients are frequently young, with 79% of patients being younger than 35 years old [72].

Clinical presentation consists in a combination of respiratory (cough, dyspnea, and chest pain), gastrointestinal, and constitutional symptoms. The suspension of vaping and steroid therapy rapidly lead to clinical improvement and recovery [70, 73].

EVALI diagnosis is strictly dependent on a recent history of vaping (within 90 days) associated with acute respiratory symptoms, CT lung abnormalities, and exclusion of other possible causes [70].

CT demonstrates various patterns of lung involvement, the most frequent being DAD and OP [73].

CT findings in EVALI are multiple, bilateral, hazy GGO centrally distributed with subpleural sparing, evident along both the chest wall and mediastinum surface. Areas of consolidations can be present but generally with a lesser extent than GGO.

Sometimes, pleural effusion and interlobular septal thickening occur, resulting in a crazy paving pattern. Centrilobular nodules are frequent, typically showing an upper lobe distribution just like the majority of the inhalation injuries [70, 71, 73].

## Drug-induced lung injury (DLI)

Adverse drug reactions, including DLI, are a major cause of morbidity and mortality, accounting for 5% of hospitalisations [74]. DLI can occur after the assumption of multiple drugs (more rarely after discontinuation of therapy) in the form of various patterns of lung damage. Immunosuppressive drugs and chemotherapies are the most frequently associated with DLI.

The non-specificity of symptoms and imaging findings implies an integration of clinical, laboratory, and imaging information to reach the diagnosis [74, 75]. DLI is a diagnosis of exclusion, based on the temporal relationship between the introduction of a suspected drug and the onset symptoms and imaging lung abnormalities, excluding other possible causes.

**Fig. 9:**
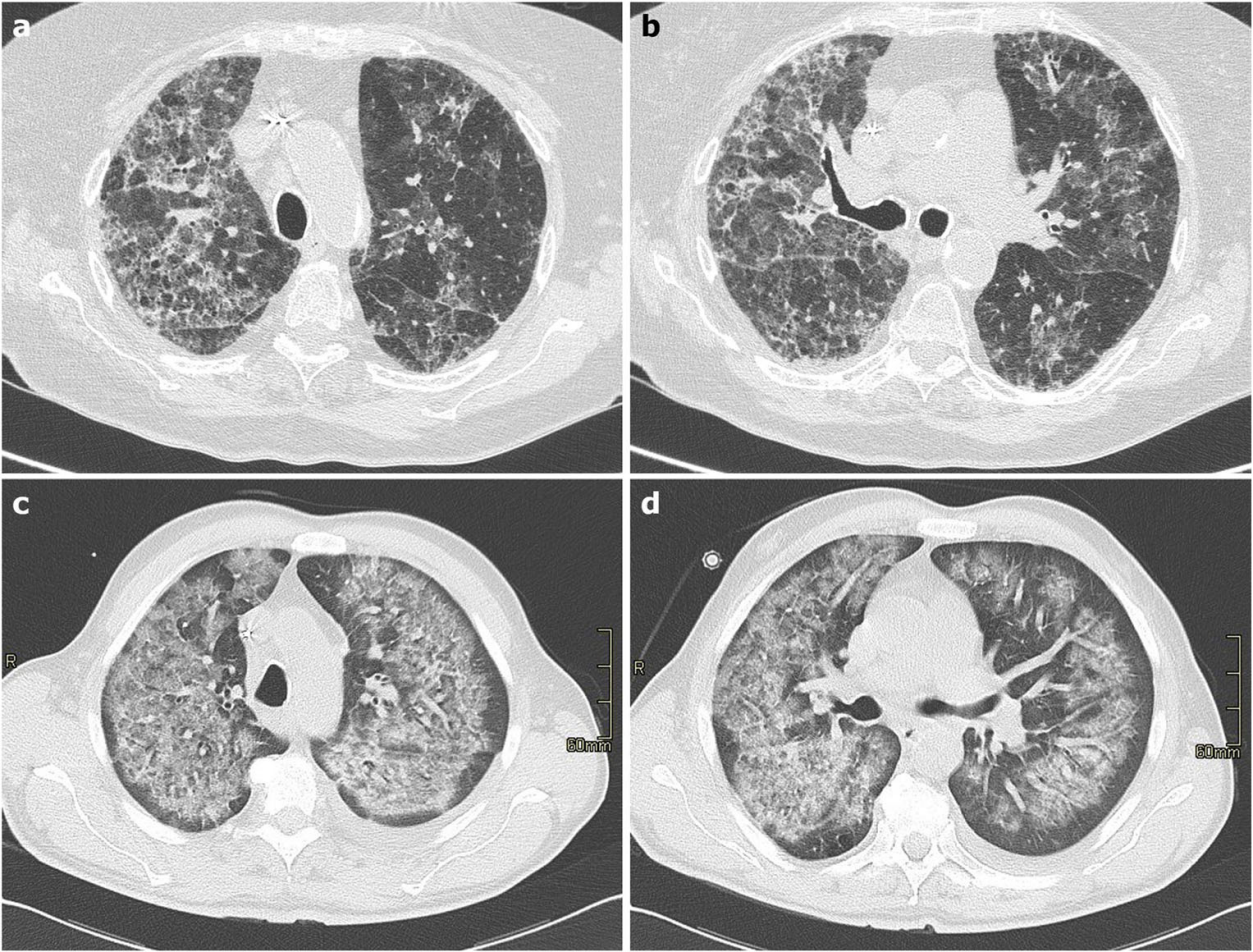
**a**–**d** Axial HRCT images of two patients who developed drug-induced lung injury secondary to amiodarone (**a**–**b**) and to anthracyclines (**c**–**d**) therapy, show bilateral patchy ground-glass opacities, consolidations, and fibrotic septal thickening (**a**–**b**); and extensive bilateral ground-glass opacities involving all lobes with subpleural sparing (**c**–**d**)

Four CT patterns have been described as the most common in DLI: a) HP pattern, frequently associated to a low dose methotrexate treatment, characterized by patchy or diffuse GGO, small poorly-defined centrilobular nodules without architectural distortion; b) DAD, the most severe form of DLI, often secondary to cytotoxic chemotherapeutic agents leading to diffuse GGO and consolidation with septal thickening and fibrotic parenchymal distortion; c) NSIP, with consolidation/GGO in a peri-broncho-vascular distribution and subpleural sparing, and d) OP consisting in multiple, non-segmental, subpleural consolidations with or without the reverse halo sign [74, 75] (Fig. 9).

Sometimes there is not one specific CT pattern, with most common findings being bilateral diffuse GGO and/or consolidations, with or without septal thickening [74].

## Others

Finally, it is worthwhile mentioning that pulmonary contusion, which may occur in case of chest trauma, and neurogenic pulmonary edema, that can be found after a significant central nervous system insults, can show similar CT appearance to that of COVID-19 pneumonia. However, the specific clinical setting generally helps identifying the correct diagnosis.

## Conclusions

Chest-CT is a sensitive diagnostic technique that can indicate COVID-19 infection in a compatible clinical setting. However, imaging features of COVID-19 pneumonia, most frequently characterized by bilateral GGO in a patchy or lower lobes prevalent distribution, are non-specific and can be observed in many different lung diseases, infectious or not, which should always be considered in the differential diagnosis especially when COVID-19 prevalence in the population is low.

## References

[1] Song F, Shi N, Shan F, Zhang Z, Shen J, Lu H, Ling Y, Jiang Y, Shi Y (2020). Emerging 2019 novel coronavirus (2019-NCoV) pneumonia. Radiology..

[2] WHO Coronavirus Disease (COVID-19) Dashboard | WHO Coronavirus Disease (COVID-19) Dashboard [Internet]. [cited 2020 Oct 11]. Available from: https://covid19.who.int/info

[3] Fang Y, Zhang H, Xie J, Lin M, Ying L, Pang P, et al. Sensitivity of chest CT for COVID-19: Comparison to RT-PCR. *Vol. 296, Radiology.* Radiological Society of North America Inc.; 2020. p. E115–7 10.1148/radiol.2020200432 PMC7233365 32073353

[4] Huang P, Liu T, Huang L, Liu H, Lei M, Xu W, et al. Use of chest CT in combination with negative RT-PCR assay for the 2019 novel coronavirus but high clinical suspicion. *Vol. 295, Radiology.* Radiological Society of North America Inc.; 2020. p. 22–3 10.1148/radiol.2020200330 PMC7233360 32049600

[5] Xie X, Zhong Z, Zhao W, Zheng C, Wang F, Liu J (2020). Chest CT for typical coronavirus disease 2019 (COVID-19) pneumonia: relationship to negative RT-PCR testing. Radiology..

[6] Kim H, Hong H, Ho YS (2020). Diagnostic performance of ct and reverse transcriptase polymerase chain reaction for coronavirus disease 2019: a meta-analysis. Radiology..

[7] Ai T, Yang Z, Hou H, Zhan C, Chen C, Lv W, Tao Q, Sun Z, Xia L (2020). Correlation of chest CT and RT-PCR testing for coronavirus disease 2019 (COVID-19) in China: a report of 1014 cases. Radiology..

[8] Litmanovich DE, Chung M, R. R. Kirkbride, Kicska G, P. J. Kanne. Review of Chest Radiograph Findings of COVID-19 Pneumonia and Suggested Reporting Language. *J Thorac Imaging.* 2020; 10.1097/RTI.0000000000000541 32520846

[9] Rubin GD, Ryerson CJ, Haramati LB, Sverzellati N, Kanne JP, Raoof S, Schluger NW, Volpi A, Yim JJ, Martin IBK, Anderson DJ, Kong C, Altes T, Bush A, Desai SR, Goldin J, Goo JM, Humbert M, Inoue Y, Kauczor HU, Luo F, Mazzone PJ, Prokop M, Remy-Jardin M, Richeldi L, Schaefer-Prokop CM, Tomiyama N, Wells AU, Leung AN (2020). The role of chest imaging in patient management during the COVID-19 pandemic: a multinational consensus statement from the Fleischner society. Chest..

[10] Hansell DM, Bankier AA, MacMahon H, McLoud TC, Müller NL, Remy J. Fleischner Society: Glossary of terms for thoracic imaging. *Vol. 246, Radiology.* Radiology; 2008. p. 697–722 10.1148/radiol.2462070712 18195376

[11] Han R, Huang L, Jiang H, Dong J, Peng H, Zhang D (2020). Early clinical and CT manifestations of coronavirus disease 2019 (COVID-19) pneumonia. Am J Roentgenol.

[12] Bai HX, Hsieh B, Xiong Z, Halsey K, Choi JW, Tran TML, Pan I, Shi LB, Wang DC, Mei J, Jiang XL, Zeng QH, Egglin TK, Hu PF, Agarwal S, Xie FF, Li S, Healey T, Atalay MK, Liao WH (2020). Performance of radiologists in differentiating COVID-19 from non-COVID-19 viral pneumonia at chest CT. Radiology..

[13] Simpson S, Kay FU, Abbara S, Bhalla S, Chung JH, Chung M, et al. Radiological Society of North America Expert Consensus Statement on Reporting Chest&ic; CT Findings Related to COVID-19. Endorsed by the Society of Thoracic Radiology, the American College of Radiology, and RSNA. *J Thorac Imaging.* 2020;35(4) 10.1097/RTI.0000000000000524 PMC7255403 32324653

[14] Léonard-Lorant I, Delabranche X, Séverac F, Helms J, Pauzet C, Collange O, Schneider F, Labani A, Bilbault P, Molière S, Leyendecker P, Roy C, Ohana M (2020). Acute pulmonary embolism in patients with COVID-19 at CT angiography and relationship to d-dimer levels. Radiology..

[15] Adams HJA, Kwee TC, Yakar D, Hope MD, Kwee RM. Chest CT Imaging Signature of Coronavirus Disease 2019 Infection: In Pursuit of the Scientific Evidence. *Chest.* 2020; 10.1016/j.chest.2020.06.025 PMC7314684 32592709

[16] Vilar J, Domingo ML, Soto C, Cogollos J (2004). Radiology of bacterial pneumonia. Eur J Radiol.

[17] Niederman MS, Mandell LA, Anzueto A, Bass JB, Broughton WA, Campbell GD, et al. Guidelines for the management of adults with community-acquired pneumonia diagnosis, assessment of severity, antimicrobial therapy, and prevention. *Vol. 163, American Journal of Respiratory and Critical Care Medicine.* American Lung Association; 2001. p. 1730–54 10.1164/ajrccm.163.7.at1010 11401897

[18] Wagner AL, Szabunio M, Hazlett KS, Wagner SG (1998). Radiologic manifestations of round pneumonia in adults. Am J Roentgenol.

[19] Lim WS, Macfarlane JT, Boswell TCJ, Harrison TG, Rose D, Leinonen M, Saikku P (2001). Study of community acquired pneumonia aetiology (SCAPA) in adults admitted to hospital: implications for management guidelines. Thorax..

[20] Marik PE (2001). Aspiration pneumonitis and aspiration pneumonia. N Engl J Med.

[21] Höffken G, Niederman MS. Nosocomial pneumonia: The importance of a de-escalating strategy for antibiotic treatment of pneumonia in the ICU. *Vol. 122, Chest.* American College of Chest Physicians; 2002. p. 2183–96 10.1378/chest.122.6.2183 12475862

[22] Chastre J, Fagon JY. Ventilator-associated pneumonia. *Vol. 165, American Journal of Respiratory and Critical Care Medicine.* American Thoracic Society; 2002. p. 867–903 10.1164/ajrccm.165.7.2105078 11934711

[23] Koo HJ, Lim S, Choe J, Choi SH, Sung H, Do KH. Radiographic and CT features of viral pneumonia. *Vol. 38, Radiographics.* Radiological Society of North America Inc.; 2018. p. 719–39 10.1148/rg.2018170048 29757717

[24] Cereser L, Dallorto A, Candoni A, Volpetti S, Righi E, Zuiani C, et al. *Pneumocystis jirovecii* pneumonia at chest High-resolution Computed Tomography (HRCT)in non-HIV immunocompromised patients: Spectrum of findings and mimickers. *Vol. 116, European Journal of Radiology.* Elsevier Ireland Ltd; 2019. p. 116–27 10.1016/j.ejrad.2019.04.025 31153552

[25] Yin Z, Kang Z, Yang D, Ding S, Luo H, Xiao E (2020). A comparison of clinical and chest CT findings in patients with influenza a (H1N1) virus infection and coronavirus disease (COVID-19). Am J Roentgenol.

[26] Liu M, Zeng W, Wen Y, Zheng Y, Lv F, Xiao K (2020). COVID-19 pneumonia: CT findings of 122 patients and differentiation from influenza pneumonia. Eur Radiol.

[27] Li X, Fang X, Bian Y, Lu J (2020). Comparison of chest CT findings between COVID-19 pneumonia and other types of viral pneumonia: a two-center retrospective study. Eur Radiol.

[28] Roshkovan L, Chatterjee N, Galperin-Aizenberg M, Gupta N, Shah R, Barbosa EM, et al. The Role of Imaging in the Management of Suspected or Known COVID-19 Pneumonia: A Multidisciplinary Perspective. *Ann Am Thorac Soc.* 2020 Oct 6; 10.1513/AnnalsATS.202006-600FR 33124905

[29] Kanne JP, Yandow DR, Meyer CA. Pneumocystis jiroveci pneumonia: High-resolution CT findings in patients with and without HIV infection. *Vol. 198, American Journal of Roentgenology.* AJR Am J Roentgenol; 2012 10.2214/AJR.11.7329 22623570

[30] Roux A, Gonzalez F, Roux M, Mehrad M, Menotti J, Zahar JR, et al. Update on pulmonary *Pneumocystis jirovecii* infection in non-HIV patients. *Vol. 44, Medecine et Maladies Infectieuses.* Elsevier Masson SAS; 2014. p. 185–98 10.1016/j.medmal.2014.01.007 24630595

[31] Hardak E, Brook O, Yigla M (2010). Radiological features of pneumocystis jirovecii pneumonia in immunocompromised patients with and without AIDS. Lung..

[32] Raghu G, Remy-Jardin M, Ryerson CJ, Myers JL, Kreuter M, Vasakova M, Bargagli E, Chung JH, Collins BF, Bendstrup E, Chami HA, Chua AT, Corte TJ, Dalphin JC, Danoff SK, Diaz-Mendoza J, Duggal A, Egashira R, Ewing T, Gulati M, Inoue Y, Jenkins AR, Johannson KA, Johkoh T, Tamae-Kakazu M, Kitaichi M, Knight SL, Koschel D, Lederer DJ, Mageto Y, Maier LA, Matiz C, Morell F, Nicholson AG, Patolia S, Pereira CA, Renzoni EA, Salisbury ML, Selman M, Walsh SLF, Wuyts WA, Wilson KC (2020). Diagnosis of hypersensitivity pneumonitis in adults. An official ATS/JRS/ALAT clinical practice guideline. Am J Respir Crit Care Med.

[33] Churg A, Muller NL, Flint J, Wright JL (2006). Chronic hypersensitivity pneumonitis. Am J Surg Pathol.

[34] Salisbury ML, Gu T, Murray S, Gross BH, Chughtai A, Sayyouh M, Kazerooni EA, Myers JL, Lagstein A, Konopka KE, Belloli EA, Sheth JS, White ES, Holtze C, Martinez FJ, Flaherty KR (2019). Hypersensitivity pneumonitis: radiologic phenotypes are associated with distinct survival time and pulmonary function trajectory. Chest..

[35] Mooney JJ, Elicker BM, Urbania TH, Agarwal MR, Ryerson CJ, Nguyen MLT, Woodruff PG, Jones KD, Collard HR, King TE, Koth LL (2013). Radiographic fibrosis score predicts survival in hypersensitivity pneumonitis. Chest..

[36] MB B, TJ F, JR G, B G, WD T. Acute fibrinous and organizing pneumonia: a histological pattern of lung injury and possible variant of diffuse alveolar damage. *Arch Pathol Lab Med.* 2002;126(9) 10.5858/2002-126-1064-AFAOP 12204055

[37] Travis WD, Costabel U, Hansell DM, King TE, Lynch DA, Nicholson AG (2013). An official American Thoracic Society/European Respiratory Society statement: update of the international multidisciplinary classification of the idiopathic interstitial pneumonias. Am J Respir Crit Care Med.

[38] Gomes R, Padrão E, Dabó H, Soares Pires F, Mota P, Melo N, et al. Acute fibrinous and organizing pneumonia: A report of 13 cases in a tertiary university hospital. *Med (United States).* 2016 Jul 1;95(27) 10.1097/MD.0000000000004073 PMC5058823 27399094

[39] Kim JY, Doo KW, Jang HJ (2018). Acute fibrinous and organizing pneumonia: imaging features, pathologic correlation, and brief literature review✰. Radiol Case Reports.

[40] Johkoh T, Fukuoka J, Tanaka T (2015). Rare idiopathic intestinal pneumonias (IIPs) and histologic patterns in new ATS/ERS multidisciplinary classification of the IIPs. Eur J Radiol.

[41] Bernheim A, McLoud T. A review of clinical and imaging findings in eosinophilic lung diseases. *Vol. 208, American Journal of Roentgenology.* American Roentgen Ray Society; 2017. p. 1002–10 10.2214/AJR.16.17315 28225641

[42] Allen JN, Pacht ER, Gadek JE, Davis WB (1989). Acute eosinophilic pneumonia as a reversible cause of noninfectious respiratory failure. N Engl J Med.

[43] De Giacomi F, Decker PA, Vassallo R, Ryu JH (2017). Acute eosinophilic pneumonia: correlation of clinical characteristics with underlying cause. Chest..

[44] Komiya K, Teramoto S, Kawashima M, Kurosaki Y, Shoji S, Hebisawa A (2010). A case of acute eosinophilic pneumonia following short-term passive smoking:an evidence of very high level of urinary cotinine. Allergol Int.

[45] Buchheit J, Eid N, Rodgers G, Feger T, Yakoub O. Acute eosinophilic pneumonia with respiratory failure: A new syndrome? In: *American Review of Respiratory Disease.* Am Rev Respir Dis; 1992. p. 716–8 10.1164/ajrccm/145.3.716 1546855

[46] Allen J. Acute eosinophilic pneumonia. *Vol. 27, Seminars in Respiratory and Critical Care Medicine.* Semin Respir Crit Care Med; 2006. p. 142–7 10.1055/s-2006-939517 16612765

[47] Rhee CK, Min KH, Yim NY, Lee JE, Lee NR, Chung MP, Jeon K (2013). Clinical characteristics and corticosteroid treatment of acute eosinophilic pneumonia. Eur Respir J.

[48] De Giacomi F, Vassallo R, Yi ES, Ryu JH (2018). Acute eosinophilic pneumonia. Am J Respir Crit Care Med.

[49] Suzuki Y, Suda T. Eosinophilic pneumonia: A review of the previous literature, causes, diagnosis, and management. *Vol.* 68*, Allergology International.* Japanese Society of Allergology; 2019. p. 413–9 10.1016/j.alit.2019.05.006 31253537

[50] Lara AR, Schwarz MI. Diffuse alveolar hemorrhage. *Vol. 137, Chest.* American College of Chest Physicians; 2010. p. 1164–71 10.1378/chest.08-2084 20442117

[51] Lichtenberger JP, Digumarthy SR, Abbott GF, Shepard JAO, Sharma A (2014). Diffuse pulmonary hemorrhage: clues to the diagnosis. Curr Probl Diagn Radiol.

[52] Zamora MR, Warner ML, Tuder R, Schwarz MI (1997). Diffuse alveolar hemorrhage and systemic lupus erythematosus: clinical presentation, histology, survival, and outcome. Medicine (Baltimore).

[53] Gluecker T, Capasso P, Schnyder P, Gudinchet F, Schaller MD, Revelly JP, Chiolero R, Vock P, Wicky S (1999). Clinical and radiologic features of pulmonary edema. Radiographics..

[54] Hewitt MG, Miller WT, Reilly TJ, Simpson S (2014). The relative frequencies of causes of widespread ground-glass opacity: a retrospective cohort. Eur J Radiol.

[55] Nowers K, Rasband JD, Berges G, Gosselin M (2002). Approach to ground-glass opacification of the lung. Semin Ultrasound CT MRI.

[56] Klimek M. Pulmonary lymphangitis carcinomatosis: systematic review and meta-analysis of case reports, 1970–2018. *Vol. 131, Postgraduate Medicine.* Taylor and Francis Inc.; 2019. p. 309–18 10.1080/00325481.2019.1595982 30900501

[57] Bruce DM, Heys SDEO (1996). Lymphangitis carcinomatosa: a literature review. J R Coll Surg Edinb.

[58] Digumarthy SR, Fischman AJ, Kwek BH, Aquino SL (2005). Fluorodeoxyglucose positron emission tomography pattern of pulmonary lymphangitic carcinomatosis. J Comput Assist Tomogr.

[59] Moubax K, Wuyts W, Vandecaveye V, Prenen H. Pulmonary lymphangitic carcinomatosis as a primary manifestation of gastric carcinoma in a young adult: A case report and review of the literature. *Vol. 5, BMC Research Notes.* BMC Res Notes; 2012 10.1186/1756-0500-5-638 PMC3519516 23158653

[60] Brander L, Reil A, Bux J, Taleghani BM, Regli B, Takala J (2005). Severe transfusion-related acute lung injury. Anesth Analg.

[61] Triulzi DJ (2009). Transfusion-related acute lung injury: current concepts for the clinician. Anesth Analg.

[62] Antoniou KM, Wells AU. Acute exacerbations of idiopathic pulmonary fibrosis. *Vol. 86, Respiration.* Respiration; 2013. p. 265–74 10.1159/000355485 24157720

[63] Kolb M, Bondue B, Pesci A, Miyazaki Y, Song JW, Bhatt NY, et al. Acute exacerbations of progressive-fibrosing interstitial lung diseases. *Vol. 27, European Respiratory Review.* European Respiratory Society; 2018 10.1183/16000617.0071-2018 PMC9488799 30578331

[64] Park IN, Kim DS, Shim TS, Lim CM, Do LS, Koh Y (2007). Acute exacerbation of interstitial pneumonia other than idiopathic pulmonary fibrosis. Chest..

[65] Kim DS, Park JH, Park BK, Lee JS, Nicholson AG, Colby T (2006). Acute exacerbation of idiopathic pulmonary fibrosis: frequency and clinical features. Eur Respir J.

[66] Churg A, Wright JL, Tazelaar HD. Acute exacerbations of fibrotic interstitial lung disease. *Vol. 58, Histopathology.* Histopathology; 2011. p. 525–30 10.1111/j.1365-2559.2010.03650.x 20854464

[67] Collard HR, Moore BB, Flaherty KR, Brown KK, Kaner RJ, King TE, et al. Acute exacerbations of idiopathic pulmonary fibrosis. *Vol.* 176*, American Journal of Respiratory and Critical Care Medicine.* Am J Respir Crit Care Med; 2007. p. 636–43 10.1164/rccm.200703-463PP PMC2094133 17585107

[68] Akira M, Kozuka T, Yamamoto S, Sakatani M (2008). Computed tomography findings in acute exacerbation of idiopathic pulmonary fibrosis. Am J Respir Crit Care Med.

[69] Fujimoto K, Taniguchi H, Johkoh T, Kondoh Y, Ichikado K, Sumikawa H, Ogura T, Kataoka K, Endo T, Kawaguchi A, Müller NL Acute exacerbation of idiopathic pulmonary fibrosis: High-resolution CT scores predict mortality. *Vol. 22, European Radiology.* Eur Radiol; 2012. p. 83–92 10.1007/s00330-011-2211-6 21822949

[70] Kligerman S, Raptis C, Larsen B, Henry TS, Caporale A, Tazelaar H, et al. Radiologic, pathologic, clinical, and physiologic findings of electronic cigarette or vaping product use-associated lung injury (EVALI): Evolving knowledge and remaining questions. *Vol. 294, Radiology.* Radiological Society of North America Inc.; 2020. p. 491–505 10.1148/radiol.2020192585 31990264

[71] Henry TS, Kligerman SJ, Raptis CA, Mann H, Sechrist JW, Kanne JP (2020). Imaging findings of vaping-associated lung injury. Am J Roentgenol.

[72] Siegel DA, Jatlaoui TC, Koumans EH, Kiernan EA, Layer M, Cates JE, Kimball A, Weissman DN, Petersen EE, Reagan-Steiner S, Godfred-Cato S, Moulia D, Moritz E, Lehnert JD, Mitchko J, London J, Zaki SR, King BA, Jones CM, Patel A, Delman DM, Koppaka R, Griffiths A, Esper A, Calfee CS, Hayes D, Rao DR, Harris D, Smith LS, Aberegg S, Callahan SJ, Njai R, Adjemian J, Garcia M, Hartnett K, Marshall K, Powell AK, Adebayo A, Amin M, Banks M, Cates J, al-Shawaf M, Boyle-Estheimer L, Briss P, Chandra G, Chang K, Chevinsky J, Chiang K, Cho P, DeSisto CL, Duca L, Jiva S, Kaboré C, Kenemer J, Lekiachvili A, Miller M, Mohamoud Y, Perrine C, Shamout M, Zapata L, Annor F, Barry V, Board A, Evans ME, Gately A, Hoots B, Pickens C, Rogers T, Vivolo-Kantor A, Cyrus A, Boehmer T, Glidden E, Hanchey A, Werner A, Zadeh SE, Pickett D, Fields V, Hughes M, Neelam V, Chatham-Stephens K, O’Laughlin K, Pomeroy M, Atti SK, Freed J, Johnson J, McLanahan E, Varela K, Layden J, Meiman J, Roth NM, Browning D, Delaney A, Olson S, Hodges DF, Smalley R, Lung Injury Response Clinical Working Group, Lung Injury Response Epidemiology/Surveillance Group, Lung Injury Response Clinical Working Group, Lung Injury Response Epidemiology/Surveillance Group, Council of State and Territorial Epidemiologists Vaping-Associated Pulmonary Injury (VAPI) Epidemiology Task Force (2019). Update: interim guidance for health care providers evaluating and caring for patients with suspected E-cigarette, or vaping, product use associated lung injury — United States, October 2019. MMWR Morb Mortal Wkly Rep.

[73] MacMurdo M, Lin C, Saeedan M Bin, Doxtader EE, Mukhopadhyay S, Arrossi V, et al. e-Cigarette or Vaping Product Use-Associated Lung Injury: Clinical, Radiologic, and Pathologic Findings of 15 Cases. In: *Chest.* Elsevier Inc; 2020. p. e181–7 10.1016/j.chest.2020.01.030 32505323

[74] Ellis SJ, Cleverley JR, Muller NL. Drug-induced lung disease: High-resolution CT findings. In: *American Journal of Roentgenology.* American Roentgen Ray Society; 2000. p. 1019–24 10.2214/ajr.175.4.1751019 11000156

[75] Sakai F, Johkoh T, Kusumoto M, Arakawa H, Takahashi M. Drug-induced interstitial lung disease in molecular targeted therapies: High-resolution CT findings. *Vol.* 17*, International Journal of Clinical Oncology.* Int J Clin Oncol; 2012. p. 542–50 10.1007/s10147-012-0489-2 23138271

